# High Performance Liquid Chromatography (HPLC) with Fluorescence Detection for Quantification of Steroids in Clinical, Pharmaceutical, and Environmental Samples: A Review

**DOI:** 10.3390/molecules27061807

**Published:** 2022-03-10

**Authors:** Fatima Hameedat, Sahar Hawamdeh, Soraya Alnabulsi, Aref Zayed

**Affiliations:** 1 Department of Medicinal Chemistry and Pharmacognosy, Faculty of Pharmacy, Jordan University of Science and Technology, Irbid 22110, Jordan; fjhmeedat13@ph.just.edu.jo (F.H.); smalnabulsi@just.edu.jo (S.A.); 2 School of Biomolecular and Biomedical Science, University College Dublin, Belfield, D04 V1W8 Dublin, Ireland; saharhawamdeh36@gmail.com

**Keywords:** steroids, HPLC, fluorescence, derivatization, quantification

## Abstract

Steroids are compounds widely available in nature and synthesized for therapeutic and medical purposes. Although several analytical techniques are available for the quantification of steroids, their analysis is challenging due to their low levels and complex matrices of the samples. The efficiency and quick separation of the HPLC combined with the sensitivity, selectivity, simplicity, and cost-efficiency of fluorescence, make HPLC coupled to fluorescence detection (HPLC-FLD) an ideal tool for routine measurement and detection of steroids. In this review, we covered HPLC-FLD methods reported in the literature for the steroids quantification in clinical, pharmaceutical, and environmental applications, focusing on the various approaches of fluorescent derivatization. The aspects related to analytical methodology including sample preparation, derivatization reagents, and chromatographic conditions will be discussed.

## 1. Introduction

Steroids are biologically active molecules that are available in many natural sources such as, animals, plants and fungi, in addition to being manufactured as therapeutics. Steroids share a common four-fused ring core consisting of 17 carbon atoms and vary according to functional groups attached to the core or side chains. Naturally secreted steroids are classified into steroid hormones (sex hormones, mineralo- and glucocorticoids, and anabolic steroids) and cholesterol. These compounds travel the bloodstream bound specifically or non-specifically to plasma proteins, such as albumin and binding globulins [1]. Steroids are key players in many biological, physiological, and pathological events, which are mediated after binding to their cognate tissue receptors. Most prominently, natural steroids play major roles in metabolism, signaling, immunity, reproduction, and salt-retaining activity. On the other hand, many synthetic steroids have been manufactured to mimic natural steroidal activity and have been approved to treat a wide variety of diseases including anti-inflammatory; arthritis, autoimmune disease, asthma and chronic obstructive pulmonary diseases [2,3].

**Figure 1:**
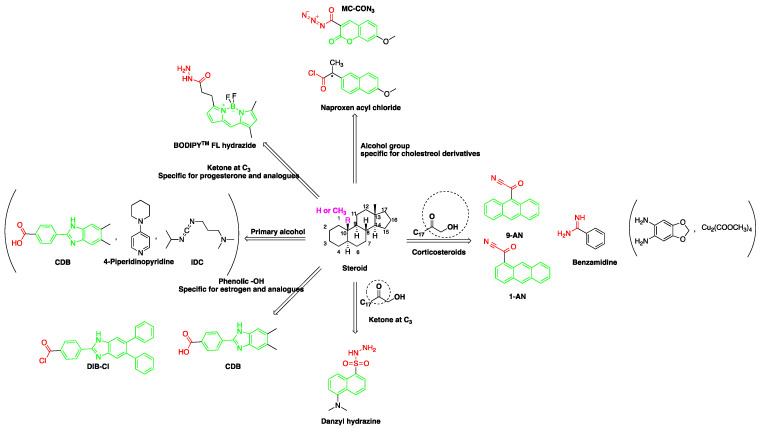
Representation of fluorescent derivatizing agents used in steroid analysis by HPLC-FLD.

Because steroids can produce biological action at very low concentrations, analytical methods must be very sensitive and precise particularly when quantifying steroid levels in biosamples. Due to the complex nature matrices of most steroid samples, the methods should also be selective to reliably quantify target steroids and resolve them from other similar endogenous compounds and interferences, and to allow for simpler extraction and pre-treatment prior to analysis. This is especially important since sample pre-treatment may lead to the loss of analyte. Steroids possess a naturally lipophilic cyclopentanoperhydrophenanthrene core (Figure 1), although their hydrophilicity is increased by reduction reactions during metabolism. Owing to this hydrophobicity, organic solvents can be used in sample preparation to extract steroids from aqueous media [4].

Commonly used methods in the analysis of steroids include immuno-assays [5,6], gas chromatography-mass spectrometry (GC-MS) [7], high-performance liquid chromatography mass spectrometry (HPLC-MS or MS/MS) [4,8], capillary electrophoresis (CE) [9], and HPLC coupled with UV [10,11,12,13] or fluorescence detection (FLD) [14]. Mass spectrometry (MS) and FLD are relevant to steroid analysis due to their inherited high sensitivity detection and low detection limit. Although both usually require incorporating a derivatization step due to weak ionization of steroids in MS and lack of fluorophores, FLD is considered a simpler and low capital cost technique.

Levels of steroids are required to be continuously monitored since any increase or decrease may be associated with major consequences on health and environment. Therefore, steroid quantification has been gaining increasing importance in disease diagnosis (e.g., increased levels of cortisol in stress and Cushing’s syndrome) [15], contamination assessment of water and food samples [16,17,18,19,20,21], detection of anabolic steroids among sportsmen due to doping [22,23,24,25,26], and detection of growth promoting steroids in animal tissue [18,25,27,28,29,30,31,32,33]. While there are several reviews on steroid quantification by HPLC methods coupled to MS and UV [8,34], to the best of our knowledge no similar reports summarizing data on HPLC-FLD methods exist. This review will discuss the applications of HPLC-FLD methods for quantifying steroids in clinical, pharmaceutical, and environmental samples. Focus will be given to the various derivatization techniques reported in the literature to introduce fluorophores to target steroids and accompanying sample preparation procedures. 

## 2. HPLC-FLD Methods

HPLC is commonly employed in the analysis of steroids as it provides an excellent separation and quantification tool. Regardless of the compound class, separation using the reversed phase mode is the method of choice in HPLC. Octadecyl silica (ODS or C_18_) columns are commonly used as the stationary phase in reversed phase-HPLC. Other materials, such as C_8_, C_2_, phenyl, amino, and cyano phases can also be used to provide different degrees of selectivity. Selectivity depends also on the composition of the mobile phase. Methanol or acetonitrile are commonly used in combination with various percentages of water to prepare the mobile phase which can be employed in the isocratic or gradient mode. Column temperature, pH of the mobile phase and its modifiers are other parameters that can be used to optimize steroids separation. Using narrow and short columns can successfully decrease the amount of solvent needed and analysis time [4].

Prior to injecting steroid-containing samples on the HPLC, a sample preparation method is commonly employed to produce the required selectivity and sensitivity. Sample pretreatment is carried out through a process of sequential steps depending on the extracted steroid and sample type/matrix. For example, biological samples are often prepared by protein precipitation (PP) and enzyme hydrolysis in order to decrease the interferences from undesired compounds such as plasma proteins and to remove the polar groups added to steroids during metabolism. In addition to PP [9], solid phase extraction (SPE) [21,35] and liquid–liquid extraction (LLE) [36,37] are commonly used extraction techniques for the analysis of steroids. Due to the continuous need for faster and simpler sample preparation procedures, other methods such as solid phase microextraction (SPME) [38], liquid-phase microextraction (LPME) [37], turbulent flow chromatography (TFC) [39], dispersive liquid–liquid microextraction (DLLME) [37], molecularly imprinted solid-phase extraction (MISPE) [40,41], restricted access material (RAM) [4,9] are also used. 

**Figure 2:**
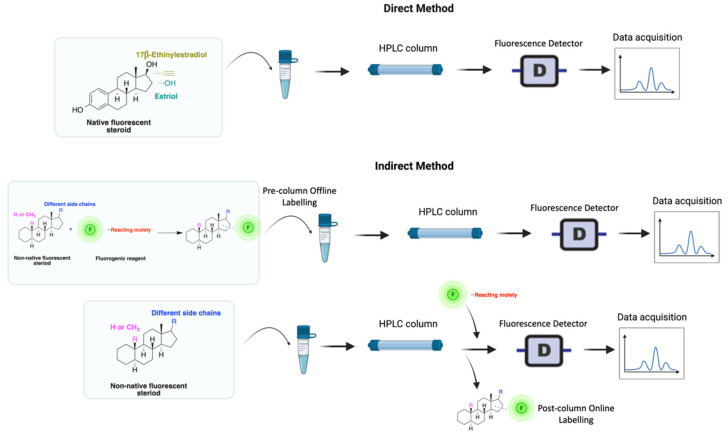
Derivatization using fluorophore-containing reagents for steroids analysis by HPLC-FLD.

HPLC-FLD detection can be carried out through direct or indirect methods, depending on the analyte’s need for extraction or/and derivatization (Figure 2). Researchers seek to develop simple methods that do not require extraction or derivatization. For example, estrogens that are native fluorescents, and certain synthetic steroids, such as the anabolic steroid trenbolone, can fluoresce and, therefore, can be quantified without derivatization. Non-native steroids and non-aromatic compounds, on the other hand, show no fluorescence characteristics and must therefore be derivatized (labeled with fluorescent moieties) prior to analysis (Figure 2).

A good derivatization procedure should be simple with high yield and minimum side products and is performed under mild conditions to avoid decomposition of the target steroid [4]. Derivatization enhances the detection of steroids and can be done by either pre-column offline labeling or post-column online labeling (Figure 2), which ideally must be rapid. These procedures can, however, be accelerated by modifying the reaction’s temperature and pH or incorporating a catalyst. Figure 1 is a representation for the most common fluorescent derivatizing pathways and their targets in steroid analysis. There are several classes of fluorescent derivatizing agents including anthracenes, coumarins and phenanthrenes. The derivatization process depends on functionalization of specific groups that are targeted in steroids structure as well as the type of derivatization agent used. For example, steroids with primary alcohol moiety, e.g., cortisone, can be targeted with a fluorescent agent in a selective reaction thus differentiating them from steroids containing secondary and tertiary alcohol groups such as prednisolone. Similarly, other steroids, such as estrogens, have specific phenolic groups, which can also be targeted by other agents. Other functionalities that react with fluorescent derivatizing agents and employed in steroid quantification are ketone and ketolic groups as will be discussed later in this review. The various derivatization procedures reported in the literature used for steroid detection by HPLC-FLD including the reagents employed and conditions used in each procedure are summarized in Table 1.

## 3. Clinical Applications of HPLC-FLD Techniques

Quantification of steroids plays an important role in the diagnosis and treatment of endocrine disorders. Diseases, such as cancer, metabolic syndromes, and neurodegenerative diseases, are associated with abnormalities in the endocrine system. Steroids also play an important role in biochemical processes, such as aging, reproduction, and metabolic pathways [76]. Furthermore, disturbances in the endocrine system due to EDC from environment and food are becoming a major clinical concern. HPLC-FLD has been employed in many research studies, both in humans and animal models, to quantify the various types of steroids and investigate their role in diseases and clinical conditions.

### 3.1. Detection of Glucocorticosteroids

Glucocorticosteroids are widely recognized as markers for adrenal activity. Cortisol can reflect the short-term changes in the activity of the hypothalamic–pituitary–adrenocortical axis (HPA axis), making it a valuable surrogate marker for stress and glucose metabolism [56].

Many methods have been developed to detect unconjugated cortisol levels in various biological samples. However, the use of certain samples, such as urine, may not be recommended as these samples may contain many interfering substances thus complicating steroids extraction procedure [55]. In addition, the concentrations of corticosteroids in urine are low compared with those found in plasma, which makes the latter a better medium for measurement. Steroids have been measured by HPLC-FLD methods for various clinical purposes, such as investigating disease courses, pathogenesis, and metabolic processes [15].

**Figure 3:**
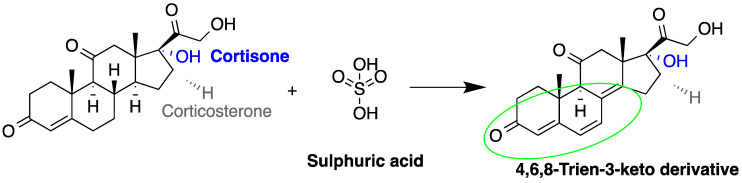
Fluorescence emission of steroids after treatment with sulfuric acid.

The detection of corticosteroids in biological samples can be done directly depending on fluorescence signal enhancement upon the treatment of samples with the deproteinizing agent (ethanol) and sulfuric acid (Figure 3). A study to quantify corticosteroids in serum samples was performed using C_18_ analytical column after treatment with ethanol and sulfuric acid [53]. Cortisol, testosterone and corticosterone emitted fluorescence, with LOD for cortisol = 0.3 pg/dL (S/N = 3). In another study, the same group measured cortisol in urine samples producing a LOD = 0.26 pg/dL (S/N = 3) [54]. Sudo et al. used post-column derivatization with sulfuric acid to measure cortisol and corticosterone in rat urine (LOD was 0.5 pmol for corticosterone at (S/N = 5)) [55]. They analyzed other corticoids, such as prednisolone, 6α-methylprednisolone, dexamethasone, and betamethasone, but with lower sensitivity. Moreover, ethyl acetate was used for extraction after sulfuric acid hydrolysis to prevent the acid from entering the HPLC system. It was noticed that the fluorescence intensities were dependent on the reactor temperature and sulfuric acid [55,77].

Since cortisol levels in hair might be used as a biomarker of chronic stress, a study developed a HPLC-FLD method to measure cortisol distribution in human hair samples. The method has achieved equal precision to mass spectrometry. Samples were prepared by pulverization and incubation in 0.1 M HCl, and extraction with ethyl acetate followed by derivatization with sulfuric acid. A detection limit of 1 pg/mg was achieved [56].

**Figure 4:**
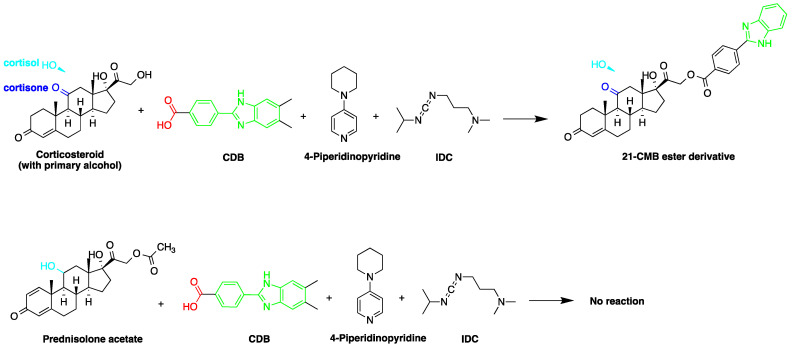
Esterification of primary alcohol functionality of cortisol and cortisone by 2-(4-carboxyphenyl)-5,6-dimethylbenzimidazole (CDB) in the presence of 4-piperidinopyridine and 1-isopropyl-3-(3-dimethylaminopropyl) carbodiimide (IDC) perchlorate. Prednisolone secondary alcohol shows no reaction.

**Figure 5:**
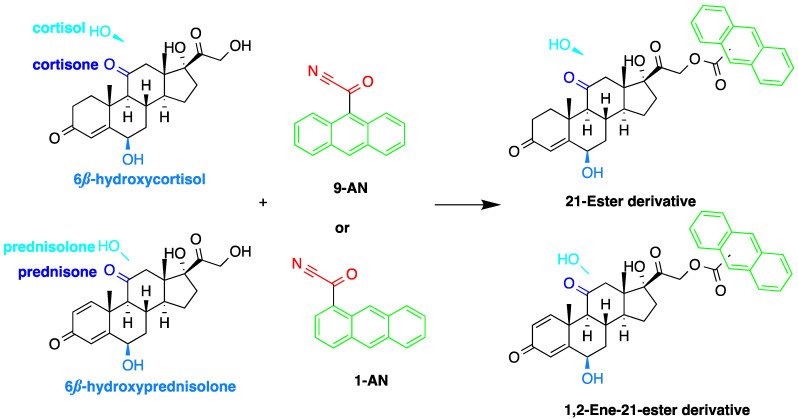
Esterification of primary alcohol functionality of cortisol and cortisone by 9-anthroyl nitrile (9-AN) or 1-anthroyl nitrile (1-AN).

Derivatization with fluorophore-containing reagents was employed in many studies to detect and quantify corticosteroids in serum and urine samples. The derivatization process depends on functionalization of specific groups in corticosteroids structure (Figure 1). The primary alcohol functionality can be esterified through its reaction with either 2-(4-carboxyphenyl)-5,6-dimethylbenzimidazole (CDB) (Figure 4), 9-anthroyl nitrile (9-AN) (Figure 5), or 1-anthroyl nitrile (1-AN) (Figure 5). These derivatization processes proved to be selective as neither secondary alcohol, nor tertiary alcohol can react with fluorophore-containing reagents (Figure 5).

The derivatization through esterification of corticosteroid primary alcohol functionality with CDB was studied by Katayama et al. [78]. The results showed that the detector response to secondary alcohols was less than one fiftieth that of the primary alcohol. However, certain secondary alcohols in steroids, such as prednisolone acetate and testosterone, and tertiary alcohols, showed no reaction. Later, Katayama et al. used human plasma to detect eight corticosteroids by derivatization with CDB to their esters in acetonitrile. The esters were separated on a reversed-phase column (Zorbax ODS) with water:methanol (25:75, *v*/*v*) containing 5 mmol/L tetramethylammonium hydrogen sulfate as a mobile phase. The LOD ranged between 0.06 and 0.3 pg per 100 µL of plasma (S/N = 3) [58].

The use of 9-AN as a derivatizing agent for corticosteroids was reported in several studies. Neufled et al. used 9-AN to derivatize the primary alcohol in C_21_ corticosteroids. The reaction was carried out at 45 °C for 2 h and the fluorescent derivatives were separated on silica stationary phase with a mixture of 2-propanol and hexane as a mobile phase in the gradient mode. The low temperature in derivatization prevented the thermal degradation of corticosteroids and avoided reaction with secondary hydroxyl groups [46,50].

Measuring endogenous glucocorticoids and their metabolites is important as disturbances in the enzyme responsible for their metabolism can cause hypertension. One study described an HPLC-FLD method using 9-AN derivatization for the determination of cortisol, cortisone, and their tetrahydro- and allo-tetrahydro-metabolites in plasma and urine [45]. Following extraction with dichloromethane and SPE, 9-AN was used to derivatize the steroids in the samples to their fluorescent products. LOD (S/N = 3:1) achieved with this method was 3.0 ng/mL for all analytes. Similarly, Shibata et al. employed 9-AN for fluorescent derivatization and developed a method to investigate cortisone levels in plasma and urine samples of renal transplant patients who received prednisolone [47]. Cortisol, cortisone, prednisolone, prednisone, 6β-hydroxycortisol, and 6β-hydroxyprednisolone were derivatized to their fluorescent esters after extraction with ethyl acetate (Figure 5). The 6β-hydroxycortisone was used as an internal standard. LODs achieved for cortisol, cortisone, prednisolone and prednisone in plasma or urine were 0.1 ng/mL, while those for 6β-hydroxycortisol and 6β-hydroxyprednisolone in plasma or urine were 0.5 ng/mL.

**Figure 6:**
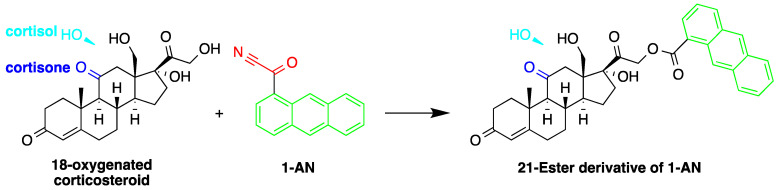
Fluorescent derivatization of 18-oxygenated corticosteroids by or 1-anthroyl nitrile (1-AN).

Similar to 9-AN, fluorescent derivatization can be achieved using 1-AN. A study described the use of 1-AN to derivatize 18-oxygenated corticosteroids: 18-hydroxycortisol, 18-hydroxycortisone and 18-oxocortisol in human urine into their fluorescent 21-anthroyl esters [43] (Figure 6). A mixture of ether and dichloromethane was used to extract the steroids, and the anthroyl derivatives were enriched by SPE using a CN cartridge column. The LOD was 0.1 pmol (S/N = 5).

**Figure 7:**
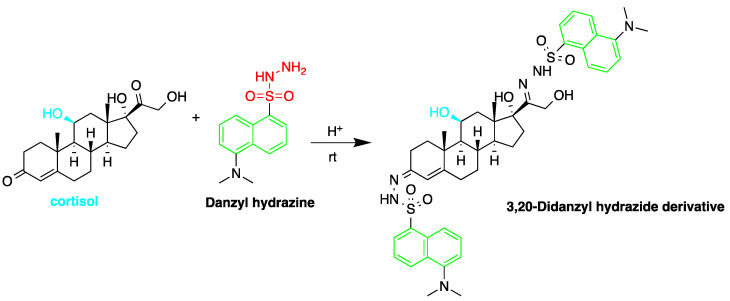
Fluorescent derivatization of carbonyl functionality of cortisol by dansyl hydrazine.

Fluorescent derivatization of corticosteroids can also be carried out using dansyl hydrazine targeting the carbonyl groups (Figure 7). In a study to determine corticosteroids in human plasma and urine samples were derivatized by dansyl hydrazine and quantified by HPLC-FLD, following extraction with methylene chloride. The linearity range of the method was between 0.5 and 60 ng of cortisol, proving it to be suitable for the routine analysis of cortisol in plasma and urine [71].

**Figure 8:**
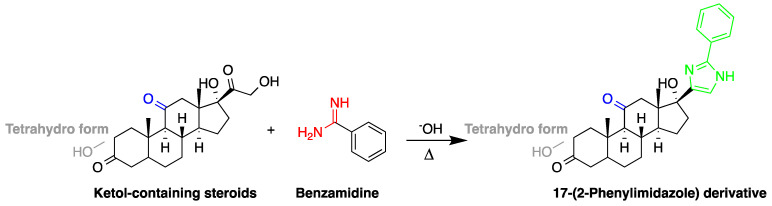
Fluorescent derivatization of ketolic-containing corticosteroids by benzamidine.

The detection of corticosteroids with ketolic groups in urine is key part in diagnostic procedure of Cushing’s syndrome. Ketol-containing corticosteroids, such as 17-hydroxycorticosteroid (breakdown product of cortisol), are usually excreted in urine as tetrahydro form making their detection using UV absorption at 240 nm not possible. To detect these steroids in urine, derivatization with amidine-containing compounds was used yielding fluorescent compounds [60] (Figure 8). Using this approach, the best sensitivity was achieved for cortisol.

**Figure 9:**
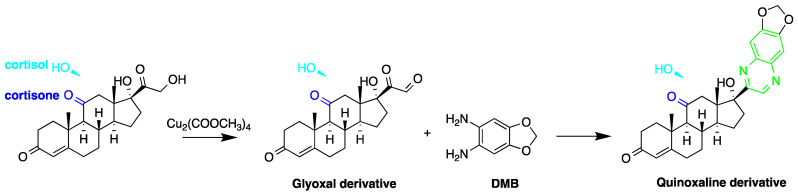
Fluorescent derivatization of ketol-containing corticosteroids to quinoxaline derivative.

The utilization of ketolic groups was also performed by Yamaguchi et al. to detect and quantify corticosteroids in urine after their conversion into fluorescent quinoxalines (Figure 9) [67]. They detected nineteen 21-hydroxycorticosteroids in human urine samples achieving LOD = 0.14–29.4 pmol/50 µL injection volume (S/N = 3). In another study by the same group, prednisolone and prednisone were quantified in plasma samples following similar derivatization procedure and analyzed by reversed-phase liquid chromatography with isocratic elution. The LOD of prednisolone and prednisone was 3 ng/mL in plasma (S/N = 3) [65].

**Figure 10:**
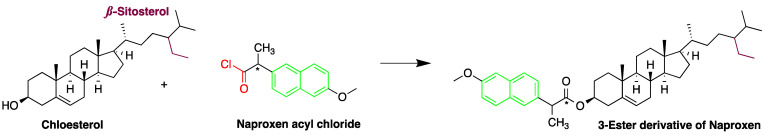
Derivatization of cholesterol by naproxen acyl chloride.

Cholesterol quantification is commonly required in the analysis of biosamples. A study described a simple and sensitive method for the determination of cholesterol and phytosterols, such as β-sitosterol in biosamples (e.g., saliva and urine matrices) and food samples (cow and soybean milk) after derivatization with naproxen acyl chloride in toluene (Figure 10). The method employed a C_8_ column with a mixture of methanol, isopropanol, and water, achieving a LOD of about 25 nM (S/N = 3). The analysis of cholesterol and sitosterol is usually time-consuming, but using the method, a relatively short period of time was required since solvent concentration, evaporation, and replacement steps were not necessary [79].

**Figure 11:**
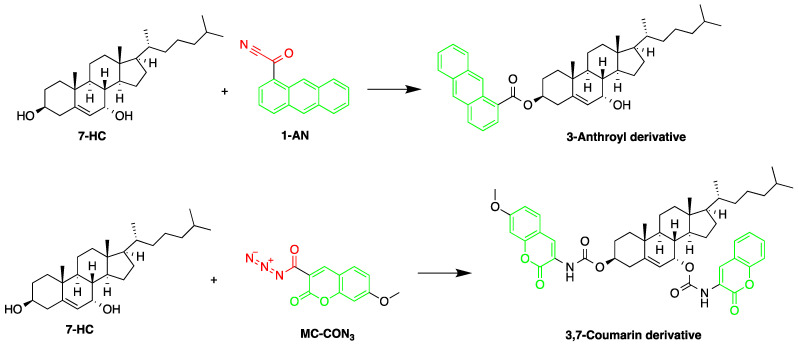
Derivatization of 7α-hydroxycholesterol (7-HC) by 1-anthroyl nitrile (1-AN) or 7-methoxycoumarin-3-carbonyl azide (MC-CON_3_).

Free 7α-hydroxycholesterol (7-HC) levels in human serum were reported to be a good indicator for bile acid synthesis. An HPLC-FLD method was developed by Saisho et al. to quantify 7-HC levels in dog plasma, with the purpose of studying the effect of cholestyramine on plasma levels of 7-HC. 7-HC was converted to its fluorescence derivative, by two procedures, after being extracted and then purified [57]. The two derivatization reagents used were 1-AN and 7-methoxycoumarin-3-carbonyl azide (MC-CON_3_). The MC-CON_3_ derivatization resulted in higher fluorescence intensity compared to 1-AN route. 1-AN produced only a C-3 fluorescent derivative due to the bulky anthracene group and steric hindrance around the C-7 position. In comparison, MC-CON_3_ yielded a double coumarin derivative at the C-3 and C-7 positions (Figure 11) producing a LOD of 4 pg (S/N = 5).

### 3.2. Detection of Steroid Hormones

**Figure 12:**
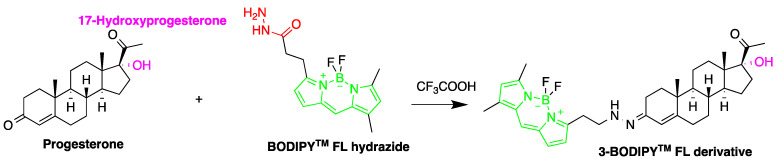
Derivatization of progesterone by 4,4-difluoro-5,7-dimethyl-4-bora-3a,4adiaza-s-indacene-3-propionohydrazide (BOD-IPYTM FL hydrazide).

Detection of steroid hormones and their metabolites are important in diagnosis of metabolic diseases. Several HPLC-FLD methods were described for the detection of steroid hormones. A study developed a method for monitoring progesterone and 17-hydroxyprogesterone in the serum from pregnant women. The quantification employed fluorescent derivatization using 4,4-difluoro-5,7-dimethyl-4-bora-3a,4adiaza-*s*-indacene-3-propionohydrazide (BODIPY^TM^ FL hydrazide) [71] (Figure 12). The derivatization was carried out in ethanol at room temperature (about 22 °C) for 15 h. This derivatization method was reported to be 50 times faster than that using dansyl hydrazine. The LODs for progesterone, 17-hydroxyprogesterone, dehydroepiandrosterone, androstenedione, testosterone and 17-methyltestosterone were in the range of 550–3700 fmol per 10 µL injection (S/N = 5).

**Figure 13:**
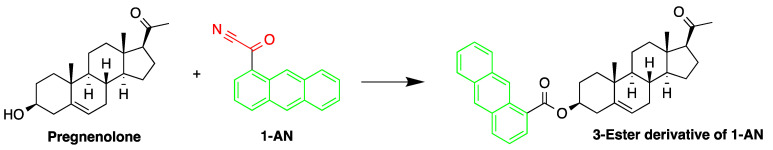
Derivatization of pregnenolone by 1-anthroyl nitrile (1-AN).

The derivatization reagent 1-AN was used by Shimada et al. to determine the endogenous steroid pregnenolone in Wistar and Sprague–Dawley rat brain samples [46]. The samples were homogenized in isotonic saline then deproteinized with methanol, before subjected to sequential steps of extraction and derivatization. The samples were derivatized with 1-AN (Figure 13) and the excess reagent was removed by purification, carried out on two successive silica gel columns. The 3β-hydroxy-16-methylpregna-5,16-dien-20-one was used as an internal standard.

Several steroids in animal tissue were detected in another study using ultraviolet, fluorometric, and electrochemical detectors [80]. The study demonstrated the selectivity of FLD in determination of estradiol by exploiting its fluorescence emission, although it was eluted with the same fraction containing other steroids and their respective metabolites. Extraction with methanol allowed the separation of acidic corticosteroids (diethylstilbestrol, estradiol, zeranol/zearalenone, and their metabolites) from their neutral anabolic counterparts (testosterone, trenbolone and progesterone).

Urine is the best source for the estimation of estrogen concentrations, since they are primarily excreted renally as glucuronides and sulfates. Previous studies used urine samples for the determination of estrogen levels in their studies, albeit with different extraction methods [42,81]. Mao et al. used chemical hydrolysis in methanol and concentrated hydrochloric acid at 70 °C for 1 h to release the conjugation before SPE sample preparation, whereas Kumar et al. used a fabric phase sorptive extraction (FBSE) procedure, which offered shorter sample preparation times and 400 times higher sorbent loading. This method achieved a lower detection limit and analysis time, proving to be greener and more economical. The LODs for β-estradiol, 17α-ethinylestradiol, and bisphenol A were 20 pg/mL, 36 pg/mL, and 42 pg/mL, respectively.

**Figure 14:**
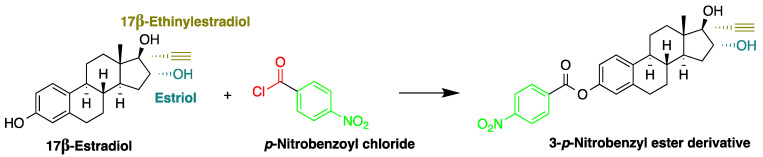
Derivatization of 17β-estradiol by *p*-nitrobenzoyl chloride.

In another study, Mao et al. used *p*-nitrobenzoyl chloride at 25 °C as a derivatization reagent, which can easily react with the hydroxyl and phenolic hydroxyl groups of organic chemicals (Figure 14) without the need for a catalyst [42]. Moreover, 4-nonylphenol, bisphenol A (BPA), 17α-ethinylestradiol, and three endogenic estrogens, including 17α-estradiol, 17β-estradiol, and estriol, were determined in urine samples collected from 20 healthy volunteers. Samples were hydrolyzed with HCl and subjected to SPE purification. Separation was performed on a C_18_ column with gradient elution using acetonitrile and water as a mobile phase. The LODs of the method were 2.7 µg/L for BPA and 17β-estradiol, 2.9 µg/L for 4-nonylphenol, 4.6 µg/L for 17α-estradiol and 17α-ethinylestradiol, and 8.3 µg/L for estriol.

A recent study examined the determination of estrone (E1), 17β-estradiol (E2), and estriol (E3) and their conjugated metabolites levels in cow and river buffalo meat [32]. Samples were extracted with methanol, enzymatically deconjugated and purified by C_18_ SPE before they are analyzed by HPLC-FLD. The effect of temperature on the studied steroid concentrations was also investigated and it was shown that heating processes was not able to significantly affect the level of phenolic estrogens in meat.

Estrogens in human urine samples were determined by HPLC-FLD following vortex-assisted dispersive liquid–liquid microextraction (VA-DLLME). Fine droplets of nonanoic acid floating on the top of sample solution were used to extract the estrogens using vortex-mix to assist the dispersion of the extraction solvent into the aqueous sample. Derivatization was not required in this work as the high extraction efficiency of DLLME improved the sensitivity and shortened the analysis time. LOD values were 0.01 ng/ml for E3, 0.01 ng/mL for βE2, and 0.06 ng/mL for E1, respectively [82].

The abuse of anabolic steroids has gained worldwide concerns, as frequent high doses can irreversibly affect the endocrine system, mineral metabolism, and may result in hepatic carcinomas [26]. Therefore, several committees have prohibited or imposed strict rules on their use for illegal purposes, such as increasing performance activity for athletes and stimulating meat production in cattle and poultry. Amin et al. used micellar chromatography with the detergent solution as the mobile phase to detect testosterone and bolasterone in human urine samples [26]. The method made use of fluorescence by means of energy transfer from the aromatic carbonyls in the anabolic steroids to the terbium ion in micellar media. No sample preparation was required and urine samples were injected directly onto the HPLC column. Excitation of terbium by means of energy transfer from steroids resulted in 183-fold fluorescence enhancement. The detection limits were 10 ng/200 µL injection volume for testosterone and 2 ng/200 µL for bolasterone.

### 3.3. Detection of Endocrine Disruptive Chemicals (EDCs)

The analysis and detection of steroid blood levels is vital for the investigation of food safety and effect on health [42,83]. Many studies have reported the presence of synthetic steroids in our daily food, both from plant and animal sources [61,84,85,86]. BPA, an industrial chemical, was found in biological fluids because of its ability to leach into food or liquids or through dental sealants into patient’s saliva [32,42,61,62,63]. One of the reasons behind their existence is that farmers increase their profit by using endocrine disruptive chemicals (EDCs) to support the feed conversion and growth rate in animals. Substances with hormonal actions are prohibited in the European community for use in animals intended for meat production due to their possible toxic effects on public health [83].

When bound to human estrogen receptors, they can stimulate the transcriptional activity of various estrogen receptor subtypes. The increase in levels of estrogen may be linked to the possibility of cancer occurrence among meat consumers [85].

In addition, studies have proved the ability of BPA to cross the placenta and blood brain barrier, in turn affecting the endocrine organs in animals and humans. This includes increasing prostate size, decreasing the number of produced sperms, and causing early puberty in females, in addition to effects on sexual differentiation [62,87]. Therefore, several HPLC-FLD methods were developed and used for quantification of EDC’s.

A sensitive HPLC method was developed for quantification of BPA and eight compounds of alkylphenol [59]. The alkylphenols assessed were; 4-*sec*-butylphenol, 2-*tert*-butylphenol, 3-*tert*-butylphenol, 4-*tert*-butylphenol, 4-n-pentylphenol, 4-*tert*-pentylphenol, 4-n-hexylphenol, and 4-n-heptylphenol. Derivatization was done using 2-(4-carboxyphenyl)-5,6-dimethylbenzimidazole (CDB) at 40 °C for 60 min. A C_18_ stationary phase was used for separation. The reported detection limits were in the range of 0.1–10.0 pg/mL. The method was applied to determination of bisphenol A in mother and infant rat serum samples.

**Figure 15:**
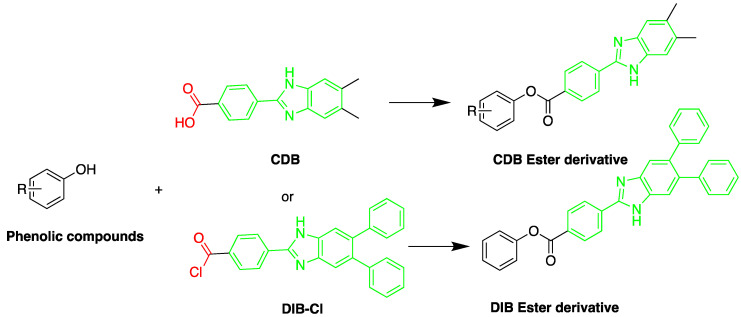
Derivatization of alkyl phenols by 2-(4-carboxyphenyl)-5,6-dimethylbenzimidazole (CDB) or 4-(4,5-diphenyl-1*H*-imidazol-2-yl) benzoyl chloride (DIB-Cl).

The fluorescent reagent 4-(4,5-diphenyl-1*H*-imidazol-2-yl) benzoyl chloride (DIB-Cl) (Figure 15) was used by Sun et al. to detect EDC’s. To the evaporated sample residue, DIB-Cl suspension in acetonitrile and triethylamine in acetonitrile were added and reacted at room temperature [63].

The study determined BPA in rat brain samples using HPLC-FLD coupled with a microdialysis [63]. A microdialysis probe was inserted into the hypothalamus of rat brains and artificial cerebrospinal fluid was used for perfusion. After the administration of a single intravenous or oral dose of BPA, concentrations were monitored in brain and plasma for 8 h. The obtained data proved that BPA could penetrate the blood brain barrier. The LOD of BPA was 0.3 ppb in 60 µL brain microdialysate at (S/N = 3).

Kuroda et al. used a similar method for determination of BPA in human blood serum and ascitic fluid samples. Samples were extracted by LLE using chloroform [62]. The LOD of BPA for both samples was 0.04 ppb at (S/N = 3). Human breast milk was also used to investigate the presence of BPA employing DIB-Cl as a fluorescent derivatizing agent [61]. Two steps of LLE were applied, and two C_18_ columns were used to separate DIB-BPA from the endogenous material in breast milk. The detection limit in 23 samples of healthy lactating women was 0.11 ng/mL at (S/N = 3).

The chromatographic conditions, sample preparation and detection limits of the HPLC-FLD methods that were employed in the steroid studies reviewed in this article are summarized and presented in Table 2.

## 4. Pharmaceutical Applications

Due to its simplicity and versatility, HPLC has become a cornerstone tool in pharmaceutical and biomedical analysis. HPLC is routinely used in drug discovery, development, and manufacturing, and routine assessment for the identification and quantification of drugs, both as active pharmaceutical ingredients and within their formulations [106]. In addition, it is essential for carrying out product characterizations, including assaying active pharmaceutical ingredients and profiling impurities [107], as well as degradation products generated by accelerated aging [108]. Moreover, the development of formulations requires studying dissolution properties, stability, and content uniformity of solid dosage forms, as well as conducting assays for the pharmaceutical formulations all of which are carried out using HPLC [106,109]. Although UV is the common detector for these applications, FLD was employed in several assays particularly in steroid analysis due to its sensitivity and selectivity as discussed below.

**Figure 16:**
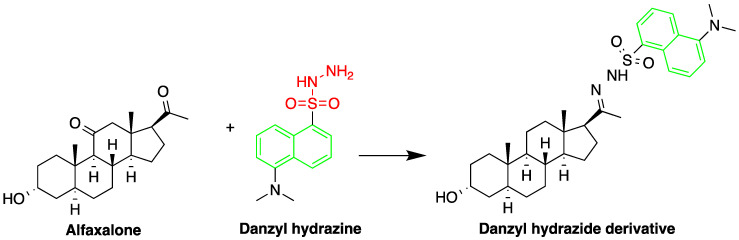
Derivatization of alfaxalone by dansyl hydrazine.

One area employing HPLC-FLD for steroid detection is investigating their pharmacokinetic–pharmacodynamic (PK–PD) properties. Vesser et al. [69] described HPLC-FLD method for quantification of the neurosteroids alfaxalone and pregnanolone in plasma samples (Figure 16). The method involved sample pretreatment with acetonitrile to precipitate plasma proteins, derivatization with dansyl hydrazine, and LLE with dichloromethane. Analysis was done using isocratic reverse phase HPLC on a 3 µM Microsphere C_18_ column. The limit of detection using 50 µL plasma sample was 10 ng/mL.

Butane acid-(5-androsten-17-one-3ß-ol)-diester (A1998), a novel dehydroepiandrosterone (DHEA) derivative antiarrhythmic steroid was successfully quantified by HPLC-FLD. Although LC–MS has the required sensitivity for quantifying low levels of steroids, A1998 is not ionizable and hard to aerosolize and therefore was not suitable for MS detection. HPLC-FLD was therefore ideal to study its PK–PD properties in rat plasma. A mixture of ZnSO_4_ solution and acetonitrile were used for sample preparation to precipitate sample plasma proteins [70] and dansyl hydrazine was used as a derivatizing agent using trifluoroacetic acid (TFA) as a catalyst to enhance the yield. A C_18_ reverse-phase column (150 mm × 4.6 mm, 5 mm) was used for the analysis and the limit of detection was 25 ng/mL (S/N = 10) using 200 μL of plasma.

Another area employing HPLC-FLD for detection of steroids is for development and characterization of drug formulation. In a recent study HPLC-FLD was used to characterize polypeptide-corticosteroid conjugates as a topical treatment approach to treat psoriasis [110]. The study successfully synthesized and characterized a pH-responsive biodegradable poly-L-glutamic acid-fluocinolone acetonide conjugate as a controlled release treatment to reduce skin inflammation. The method employed C-18 LiChrospher analytical column (125 × 4.0 mm), with a flow rate of 1 mL/min, the using a mobile phase of H_2_O (1% orthophosphoric acid)/ACN (60/40) [110].

Due to the low administered dose of ethinyl estradiol, which can be as low as 10 µg/mL, methods employ derivatization or require composite of samples of up to 10 tablets to improve the quantification limits in performing uniformity and dissolution tests [26,111]. Nonetheless, ethinyl estradiol is a native fluorescent, and it is therefore simpler to measure using direct methods. Strusiak et al. used reversed phase HPLC-FLD with a mobile phase of 0.05 M aqueous KH_2_PO_4_-methyl alcohol (2:3) [112] for measuring ethinyl estradiol in tablets. The method required minimal sample preparation and can be applied to measure ethinyl estradiol in combination with other compounds, such as methyl testosterone and progesterone.

Glowka et al. measured triamcinolone (TMC) in the presence of endogenous corticosteroids in human plasma, after its administration as tablets to healthy volunteers [88]. The samples were subjected to SPE before derivatization with 9-anthroyl nitrile (9-AN) in a basic mixture. An isocratic RP-HPLC was performed using a C_18_ column and a mixture of acetonitrile and 0.3 mM ortho-phosphoric acid as the mobile phase. The method recorded LOD of 1.0 ng/mL (S/N = 6/1).

The use of HPLC-FLD for the analysis of commercial estrogens using post-column online photochemical derivatization was investigated by Gatti et al. [90]. Separation of conjugated and unconjugated estrogens were optimized using a Phenomenex Prodigy column 5 ODS2, and a mobile phase consisting of a TEA phosphate buffer (pH 4.0; 0.05 M) and acetonitrile at different concentrations and flow rates. The addition of photochemical derivatization to the HPLC-FLD method recorded a 1000 times higher sensitivity than UV. The LODs of conjugated estrogens were between 0.03 and 0.19 pmol (S/N = 3). This method provided useful information regarding quality of estrogen in raw materials and conjugated estrogens in dosage forms.

HPLC coupled with UV/FLD was used by Arsova-Sarafinovska et al. for determination of ethinylestradiol and levonorgestrel, present at very low levels in a low-dose oral contraceptive. The method employed Purospher^®^ reversed phase column (150 × 4.0 mm I.D., particle size 5 μm) and a 47%: 53% acetonitrile: water (*v*/*v*). Drospirenone, was used as internal standard. FLD showed excellent detection limit for ethinyl estradiol (0.65 ng/mL) which was 83 times lower compared to that produced by UV [12].

Similarly, Silva et al. used HPLC coupled with UV/FLD to quantify ethinyl estradiol and the synthetic progestin drospirenone in coated tablets [73]. The method conditions were comparable to that developed by Arsova-Sarafinovska et al. but reported higher detection limits using FLD (20 ng/mL) [89]. The method was fully validated and was found suitable for routine use in quality control laboratories.

## 5. Environmental and Food Applications

Steroids can be found in various environmental samples, including water, plant, and animal samples. Water is the transporter medium and reservoir for various synthetic steroids, whereas plants were found to contain many different natural steroids [17,52,93,94,95], which can be classified according to their biological relevance. For example, plant physiological steroids can be present as hormones (e.g., brassinolide) and pheromones (e.g., antheridiol), whereas plant allelochemical substances are biologically related to animal hormones such as vertebrate hormones (progesterone) and insect hormones (ecdysone). Other plant steroids can act as protective steroids (e.g., digitoxigenin, solanidine) [51,99,113].

Exogenous estrogens can enter the human body through sewage discharge and animal waste disposal. It has been reported that the discharge of industrial and municipal wastewater can introduce up to 60% of the manufactured surfactant alkylphenol ethoxylate (APE) [42]. APE can degrade to more toxic products by sewage treatment plants (STP), such as nonylphenol (NP), octylphenol (OP), and their metabolites [28], which can cause toxicity at a low concentration range (3.7–6.0 μg/L). Sample preparation procedures and analytical methods used for analysis of steroid hormones in environmental and food samples have been recently reviewed[114].

Many studies used HPLC-FLD for the analysis of steroids in environmental samples due to its sensitivity and selectivity. Studies investigated the steroid content in samples collected from drinking water, wastewater, river water, tap surface water, and mineral and underground river water [17,20,37,52,92,93,94,95]. Steroids were also analyzed in samples taken from effluent, influent, and sediment wastewater in treatment plant [41]. Other studies have collected samples from fish, chicken, cows, river buffalos, and measured their steroid content [31,73,95].

Lu et al. have analyzed poultry litters samples to detect estrogens 17β-estradiol (E2) and 17α-ethinylestradiol (EE2). The samples were extracted with a mixture of dichloromethane and methanol followed by a clean-up procedure using normal-phase open column chromatography to remove lipid contents. The detection limits for E2 and EE2 were 4.0 μg/kg and 2.6 μg/kg, respectively [91].

In another study, a method was developed to detect four endocrine disruptors: 17-estradiol, estriol, bisphenol A, and 17-ethinylestradiol in environmental water using online monolithic SPME with HPLC-FLD [96]. The speed of the analysis was improved using the extraction medium, poly(acrylamide-vinylpyridine-*N*,*N* methylene bisacrylamide) monolith, which was synthesized inside a polyether ether ketone (PEEK) tube. The method was applied successfully to the analysis of several types of environmental water samples. Low detection limits in the range of 0.006–0.10 ng/mL (S/N = 3) were achieved for the target compounds.

Another method based on SPME monolithic extraction media coupled with HPLC-FLD was also employed by Fan et al. to determine bisphenol A and 17α-ethinylestradiol in environmental water samples. The method achieved high extraction efficiency and produced a detection limit of 0.064 and 0.12 ng/mL for bisphenol A and 17α-ethinylestradiol, respectively. The method was applied successfully to the analysis of environmental water samples from different sources demonstrating the robustness of the method in real settings [98].

Online extraction was also employed by another group using SPE with HPLC-FLD for the quantification of selected EDCs in water. Ying et al. tested three types of SPE cartridges to preconcentrate nonylphenols, octylphenols, POE(1-2) nonyl phenol and bisphenol A as well as hormone steroids estradiol, estriol, ethinylestradiol, and ethinylestradiol 3-methyl ether in deionized water. The highest recoveries were obtained with PLRP-s and PRP-1 polymer cartridges compared to C_18_ cartridges. The method was applied successfully on a river water sample spiked with the EDCs. The detection limits ranged between 20 and 50 ng/L [97].

Another recent study investigating river water samples by HPLC-FLD was carried out by Ali et al. [34]. The study employed a derivatization procedure, which targets the ethinyl group rather than the phenolic group to react with an aryl halide in the presence of palladium (Pd) and copper catalysts. The fluorescent aryl halide labeling reagent used in this reaction is 4-(4,5-diphenyl-1*H*-imidazol-2-yl) iodobenzene (DIB-I); was prepared by the same group. The method achieved a low detection limit (S/N = 3) of 7.4 ng/L [34].

Zhang et al. determined eight EDCs in wastewater using precolumn derivatization and SPE-HPLC-FLD. In the study 4-octylphenol, 4-nonylphenol, bisphenol A, diethylstilbestrol, estrone, 17α-ethinylestradiol, 17β-estradiol, and estriol were derivatized with 10-ethyl-acridone-2-sulfonyl chloride (EASC). The method was successfully applied to the determination of the EDC compounds in wastewater samples with a significant improvement in sensitivity compared to traditional HPLC methods. The LODs of the method were between 0.3 and 0.7 ng/L [52].

Four trace estrogens were determined in different environmental samples using solid–liquid extraction/auto SPE and HPLC-FLD method developed by Liu et al. The LODs of the method were down to 1.1 × 10^−2^ (estrone), 4.11 × 10^−4^ (estradiol), 5.2 × 10^−3^ (estriol), and 7.18 × 10^−3^ µg/L (17α-ethynyl estradiol)[21]. The study that quantified estrogens in samples collected at livestock farms and a major river in Northeast China, warned that high levels were observed and that estrogenic fate and contamination should be investigated in the region [21]. The same HPLC-FLD method was employed in two recent studies investigating steroid-degrading strains of bacteria as an approach for tackling increased environmental pollution of steroids [115,116].

Municipal wastewater treatment plants were sampled to investigate the presence of the steroids nonylphenol (NP), octylphenol (OP), nonylphenol polyethoxylates (NPE), 17α*-*estradiol (E2), and ethinylestradiol (EE2) [5]. SPE disks were used for extraction, followed by HPLC coupled with FLD or competitive radioimmunoassay (RIA). The recorded LODs for HPLC-FLD were 11, 2, and 52 ng/L of water for NP, OP, and NPE, respectively, but were higher than those obtained with HPLC-RIA for E2 and EE2, recorded as 107 and 53 pg/L, respectively [5]. However, safety is a major limitation for routine use of RIA.

Lima et al. developed a low-cost fast method using DLLME with HPLC-FLD for quantification of estrogens in water. The method produced high recoveries and detection limits of 2.0 ng/L and 6.5 ng/L for E2 and EE2, respectively. The method that is suitable for analyzing large number of samples and uses low amount of extraction solvent was applied successfully to the analysis of tap, surface, and waste water samples [37].

The adsorption of estrogens on different types of powdered activated carbon (PAC) was investigated by Yoon et al. using HPLC-FLD. The study demonstrated the feasibility of using PAC for removal of >99% of BPA, 17 β-estradiol (E2), and 17α-ethynyl estradiol (EE2) from raw drinking waters at least at initial concentration of 500 ng/L and higher [94]. PAC type, dosage, and the presence or absence of natural organic matter determined the percentage of steroid removal. The removal was improved by increasing both PAC dose and contact time. The HPLC-FLD method had detection limits of 0.88, 1.15, and 0.96 nM for BPA, E2, and EE2, respectively.

Kozłowska-Tylingo et al. compared the different methods and extraction procedures for the analysis of five estrogens in drinking water and wastewater samples. Extraction of 17α-ethinylestradiol, estrone, estradiol, estriol, and progesterone produced the best recoveries using extraction disks compared to C_18_ extraction columns. SPE–LC–MS/MS produced the best sensitivities compared to other methods employing UV and FLD [19].

Zhang et al. investigated ionic liquid based pretreatment for quantification of three steroids in water samples by HPLC-FLD. The sample pretreatment has the advantages of being safer and environmentally friendly since no organic solvents is used. The method based on ionic liquid-based homogeneous liquid–liquid microextraction (IF-IHLME) produced detection limits of 0.04, 0.05, and 0.05 ng/mL for 17-α-estradiol, 17-β-estradiol-benzoate, and quinestrol, respectively [101].

Estrogens and their metabolites were also quantified in a study by Guedes-Alonso et al. using molecularly imprinted (MIP) solid-phase extraction coupled with UPLC-FLD. The method was used to analyze wastewater from a veterinary hospital as well as influent and effluent samples of a wastewater treatment plant. The limits of detection were between 0.18 and 0.45 ng/mL [41].

The occurrence of trenbolone acetate metabolites, which is widely used to promote the growth of beef cattle, was evaluated in samples from a beef cattle feedlot discharge and in river water upstream and downstream from the discharge [95]. HPLC-FLD using methanol and water as the mobile phase in gradient flow and C_18_ column with dimensions of 4.6 mm × 250 mm was employed to quantify 17α- and 17β-trenbolone in the samples. The recorded LOD for was around 4 ng/L. The same metabolites were also quantified in bovine muscle, and liver samples by SPE-HPLC-FLD [33]. In the study, Yoshioka et al. examined several extraction solvents, such as methanol, diethyl ether, acetonitrile, and ethyl acetate, with the latter resulting in the cleanest extracts [33]. LODs in liver and bovine muscle samples were 0.2 and 1.0 ng/g, respectively.

Determination of natural and synthetic estrogenic compounds in dairy products was carried out using hollow fiber liquid-phase microextraction coupled to HPLC-FLD/PDA. Estriol, 17β-estradiol, 17α-estradiol, estrone, 17α-ethinylestradiol, diethylstilbestrol, dienestrol, and hexestrol) and 2-hydroxyestradiol were quantified in natural yogurt, a probiotic yogurt-type drink and cheese. The method produced LODs in the low lg/kg or lg/L range with good precision and accuracy [36].

Ultrasonic assisted dispersive liquid–liquid microextraction (UA-DLLME) method coupled with 9-phenanthreneboronic acid derivatization was used for the quantification of brassinolide a plant hormone by HPLC-FLD. Different extraction and degravitation conditions were investigated and optimized. The method was applied for brassinolide determination in *Arabidopsis thaliana*, *Daucus carota* and *Brassica campestris* L. leaves with higher sensitivity than similar reported methods. LOD of the method was 8.0 ng/L [99].

A study by Ito et al. used HPLC-FLD method for the quantification of different phytosterols and cholesterol in land plants and marine algae after derivatization with 1-anthroyl nitrile. The method used a C-30 column and an isocratic mobile phase of acetone/acetonitrile/hexane/water (71:20:4:5, *v*/*v*) at 1.0 mL min^−1^. The LODs for the different sterols were between 0.25 and 0.40 μg/mL [117].

Zearalenone an estrogenic mycotoxin that contaminates cereal crops was quantified in edible oil by an automated SPE–HPLC-FLD. Dryzmala et al. employed a SPE for reversible hydrazone formation by zearalenone and a hydrazine moiety covalently attached to a solid phase. The results of the HPLC-FLD method were found in agreement with an isotopic dilution LC–MS/MS method. The LOD of the method was 10 µg/kg [100].

Alkylphenol polyethoxylates are nonionic surfactants that act as EDCs and can exert estrogenic effects on fish [118,119]. To assess their contamination in fish and shellfish samples, Tsuda et al. developed a simple and highly sensitive HPLC method [32]. The method was used to detect 4-nonylphenol (NP), 4-nonylphenol mono-(NP1EO), diethoxylates (NP2EO), bisphenol A (BPA), 4-*tert*-butylphenol (BP), and 4-*tert*-octylphenol (OP). Acetonitrile was used for extraction, followed by clean-up with a magnesia silica gel adsorbent, Florisil PR, before analysis with HPLC-FLD. The mobile phase was water and methanol with gradient flow. The detection limit was 2 ng/g for NP, NP1EO, and NP2EO, and 1 ng/g for BPA, BP, and OP.

Similarly, Datta et al. examined fish tissue for presence of alkylphenolic compounds using an HPLC-FLD method [28]. Extraction of ground fish tissue was conducted using pressurized fluid extraction, followed by purification on amino propyl silica cartridges. Hexane and ethanol were used as the mobile phase. The recorded LOD was 4–15 ng/mL.

## 6. Conclusions

This review summarizes the various HPLC-FLD methods reported on in the literature for the quantification of steroids in clinical, pharmaceutical, food, and environmental samples. Researchers in different fields employed HPLC-FLD to meet the needs of their applications in terms of sensitivity and selectivity. Since most steroids lack inherited fluorescent properties, various fluorophore labels were introduced to target steroids through different derivatization approaches to enhance steroids quantification. Reaction schemes included in this review illustrated the chemical coupling of the fluorescent label to steroid compounds and highlighted how different steroids can be selectivity targeted depending on the functional group present in the steroid. It was shown that derivatization approaches vary according to speed, sensitivity, steroid type, the need for sample preparation/purification, and the functional group targeted, therefore offering researchers a selection of options that suit their applications. For this purpose, a table that summarizes the various derivatization approaches used and specific conditions reported on is included in this review, to help researchers quickly compare between these approaches. Another table focuses on the chromatographic and analytical method conditions, highlighting performance characteristics, such as LOD and LLOQ, and a sample matrix, which are critical for choosing the most relevant method, depending on the steroid levels and sample type. Most of the HPLC-FLD methods reported were applied in the clinical field and its related research, which is expected, due to the need for higher selectivity and sensitivity that is relevant to the complex nature of the matrix and low steroid levels of the samples. HPLC-FLD methods also found applications in the pharmaceutical and environmental fields, where sample preparation was mainly employed to enrich the low levels of steroids found in less complex sample matrices, such as water. Overall, this review provides a comprehensive summary of the various methods and procedures which are reported in the literature that can be applied to quantify steroids by HPLC-FLD.

## Figures and Tables

**Table 1:** Derivatization reagents and conditions used in steroid quantification by HPLC-FLD.

Reagent	Target Steroid	Sample Preparation and Derivatization Conditions	Reference
*p*-Nitrobenzoyl chloride 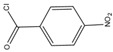	Estrogens	Sample was extracted by SPE C_18_. Residue reacted with *p*-nitrobenzoyl chloride at 25 °C, 30 min	Mao et al. 2004 [42]
1-Anthroyl nitrile (1-AN) 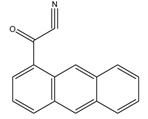	18-Oxygenated corticosteroids, 18-hydroxycortisol, 18-hydroxycortisone and 18-oxocortisol, Pregnenolone and C21 steroids; especially corticoids	Sample was extracted by SPE or LLE with a mixture of diethyl ether and dichloromethane. Extract reacted with 1-anthroyl nitrile in acetonitrile at room temperature for 10 min.	Kurosawa et al. 1995 [43] Shimada et al. 1996 [27] Shimada et al. 1991 [44]
9-Anthroyl nitrile (9-AN) 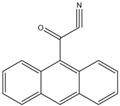	Glucocorticoids; F and E, Hydroxysteroids Corticosteroids	Sample was extracted by LLE or SPE. Extracts reacted with 9-AN in a mixture of quinuclidine and triethylamine for 30 min at RT.	Glowka et al. 2009 [45] Goto et al. 1983 [46] Shibata et al. 1997 [47] Haegele et al. 1991 [48] Kosicka et al. 2018 [49] Neufeld et al. 1998 [50] Shimada et al. 1991 [44]
2-(11H-Benzo[a]carbazole-11-yl) ethyl carbonochloridate (BCEC-Cl) 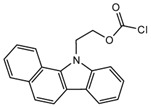	E1, E2, E3, BPA, NP, OP	Sample was extracted by DLLME. Extracts were added to BCEC-Cl in a NaHCO_3_ buffer at pH 10; it was shaken for 10 s, and then allowed to stand for 14 min at 43 °C.	Wu et al. 2015 [29]
9-Phenanthrene boronic acid 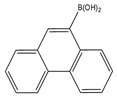	Brassinosteroids	Sample was extracted by LLE. Extracts reacted with 9-phenanthrene-boronic acid in a mixture of pyridine and acetonitrile for 10 min at 70 °C.	Gamoh and Takatsuto. 1989 [51]
10-Ethyl-acridone- 2-sulfonyl chloride (EASC) 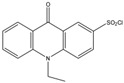	Estrogens and Biogenic Amines (aliphatic amines)	Sample was extracted by LLE or SPE. Extracts reacted with EASC in anhydrous acetonitrile and NaHCO_3_ buffer pH (10.2), for 5 min at 60 °C.	Zhang et al. 2012 [52]
Sulfuric acid 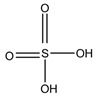	F and Corticosterone	Sample was extracted by SPE or LLE. Extracts reacted with sulfuric acid in ethanol; it was cooled in crushed ice for 15 min in the dark.	Nozaki et al. 1991 [53] Nozaki et al. 1992 [54] Sudo et al. 1990 [55]Gao et al. 2010 [56]
7-Methoxycoumarin-3-carbonyl azide (MC-CON_3_) 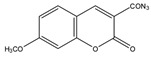	7α-hydroxycholesterol	Sample was extracted by LLE and purified by normal-phase SPE. Extracts reacted with MC-CON_3_ in ethyl acetate for 40 min at 140 °C.	Saisho et al. 1998 [57]
2-(4-Carboxyphenyl)-5,6-dimethylbenzimidazole(CDB) 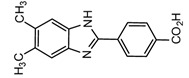	Corticosteroids, BPA and alkylphenols	Sample was extracted by LLE. 0.02% *w*/*v* CDB solution, 2.0% *w*/*v* 1-isopropyl-3-(3-dimethylaminopropyl) carbodiimide perchlorate (IDC) solution, and 0.01% *w*/*v* (10 mg/mL) 4-piperidinopyridine, for 60 min at 40 °C.	Katayama et al. 1991 [58] Katayama et al. 1992 [58] Katayama et al. 2001 [59]
Benzamidine 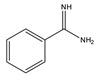	17-hydroxycorticosteroids	Sample was extracted by LLE. Extracts reacted with benzamidine in a basic medium of sodium hydroxide solution, with a mixture of propanol and water for 5 min at 95 °C.	Seki et al. 1984 [60]
4-(4,5-Diphenyl-1*H*- imidazol-2-yl) benzoyl chloride (DIB-Cl) 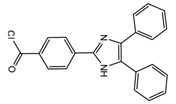	BPA	Sample was extracted by LLE or SPE. Extracts reacted with DIB-Cl in a mixture of acetonitrile and triethylamine for 20 min at RT.	Sun et al. 2004 [61] Kuroda et al. 2003 [62] Sun et al. 2002 [63]
4-(4,5-Diphenyl-1*H*-imidazol-2-yl) iodobenzene (DIB-I) 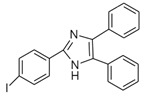	EE	Sample was extracted by the SPE disc method (C_18_ SPE disk). A total of 50 μL of the extracts reacted with 50 μL of DIB-I (3.0 mM), in a mixture of 50 μL of solution containing 0.2 mM of PdCl_2_ and 0.3 mM of CuI, and 50 μL of DIPEA (3.0 mM). The vial contents were deoxygenated by N_2_ purge for 10 sec, heated at 100 °C for 40 min, cooled, then filtered through a 0.45-μm membrane filter before injection into the HPLC-FLD system.	Ali et al. 2020 [64]
1,2-Diamino-4,5-methylenedioxybenzene (DMB) 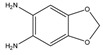	α-Dicarbonyl compounds; prednisolone, PN and 21-hydroxycorticosteroids	Sample was extracted by LLE. Extracts reacted with DMB for 40 min at 60 °C.	Yamaguchi et al. 1991 [65] Yoshitake et al. 1989 [66] Yamaguchi et al. 1989 [67]
2-(4-Carboxyphenyl)-5,6-dimethylbenzimidazole (BODIPY FL hydrazide) 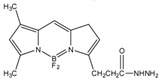	Aldehydes and ketones; progesterone, 17-hydroxyprogesterone, and other 3-keto steroids.	Sample was extracted by LLE. Extracts reacted with BODIPY FL hydrazide in ethanol for 15 h at RT.	Katayama et al. 1998 [68]
Dansyl hydrazine 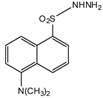	F, Butane acid-(5-androsten-17-one-3beta-ol)-diester (A1998)[69], alfaxalone and pregnanolone [70]	Sample was extracted by LLE. Extracts reacted with dansyl hydrazine in organic solvent for 30 min at RT in acidic medium.	Kawasaki et al. 1979 [71] Visser et al. 2000 [69] Peng et al. 2007 [70]
Naproxen acyl chloride in toluene 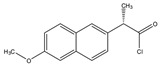	Cholesterol and sitosterol	Sample was extracted by LLE. Extracts reacted with naproxen acyl chloride in toluene, while shaking for 1.5 h at 90 °C. Diethylamine in toluene was then added to inactivate the excess naproxen acyl chloride, while shaking for 5 min at 30 °C.	Lin et al. 2007 [38]
Dansylaminophenylboronic acid 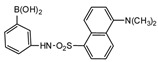	Brassinolide and castasterone	Sample was extracted by LLE. Extracts reacted with dansylaminophenylboronic acid in a mixture of pyridine and acetonitrile for 20 min at 70 °C.	Motegi et al. 1994 [72]
9-Fluorenylmethyl chloroformate (Fmoc-Cl) 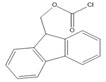	E1, E2, E3, BPA, NP, OP	Samples were extracted by MSPE. Extracts were reacted with Fmoc-Cl in NaHCO_3_ (pH = 10.5) at 60 °C for 10 min, and then added to a mixture of aqueous acetic acid and acetonitrile. The mixture was cooled to RT.	Qianyu Li et al. 2018 [73]
2-(11H-Benzo[a]carbazole-11-yl)- ethyl-4-methylbenzenesulfonate (BCETS) 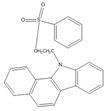	FFA	The samples were extracted by supercritical CO_2_ and organic solvent extraction. The extracted samples were derivatized in BCETS in a solution of K_2_CO_3_ at 84 °C; then the mixture was cooled down to RT and diluted with DMF.	Li et al. 2011 [74]
Benzimidazo[2, 1-b]quinazoline-12(6H)-one-5-ethylimidazole ester (BQEIC) 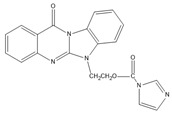	OP, NP, TBP, BPA, E1, E2, E3	The samples were extracted by LLE. BQEIC in a solvent of DMAP at 80 °C for 60 min. Finally, the mixture was cooled to RT and diluted with acetonitrile.	Liu et al. 2018 [75]

Abbreviation: solid-phase extraction (SPE), liquid–liquid extraction (LLE), 4-octylphenol (OP), 4-tert-octylphenol (4-t-OP), 4-nonylphenol (NP), 4-tert-butylphenol (TBP), bisphenol A (BPA), estrone (E1), 17β-estradiol (17β-E2), 17α-estradiol (17α-E2), estriol (E3), free fatty acids(FFA), 17α-ethinylestradiol (EE2), ethinylestradiol (EED), cortisol (F), cortisone (E), 4,40-(1,2-diethylethylene)diphenol (HEX), prednisolone (PL), prednisone (PN), 6β-hydroxycortisol (6β-OHF), 6β-hydroxyprednisolone (6β-OHP) and 6β-hydroxycortisone (6β-OHE), room temperature (RT).

**Table 2:** HPLC-FLD method conditions used for steroid detection.

Detected Steroids	Sample Type	Column Chemistry	Mobile Phase (*v*/*v*)	Derivatization Agent	Excitation (λex) and Emission Wavelengths (λem) (nm)	Extraction Method	LOD	References
PL and PN	Human plasma	C_18_	MeOH:ACN:1.0 M ammonium acetate (38:25:45)	DMB	350, 390	LLE	3 ng/mL	Yamaguchi et al. 1991 [65]
F and E	Biological samples	Keystone Hypersil	H_2_O:MeOH:ACN (50:33.3:16.7)	9-AN	305–395, 430–470	SPE	F: 50 pg E: 70 pg	Haegele et al. 1991 [48]
F	Human serum	C_18_	1: 10 mM potassium biphthalate 2: ACN Tetrahydrofuran: 19 mM potassium biphthalate (40:6:54) Both adjusted to pH 1.85 with trifluoroacetic acid	Sulfuric acid-ethanol	365, 520	-	0.30 pg/dL	Nozaki et al. 1991 [53]
F	Human urine	C_18_	Gradient of ACN: 36.4 mmol/L phosphate (45:55; pH 1.85 with trifluoroacetic acid)	Sulfuric acid-ethanol	365, 520	SPE	0.26 pg/dL	Nozaki et al. 1992 [54]
7α-Hydroxycholesterol	Dog plasma	Develosil Ph-5	Acetonitrile: Water (5:2)	1-AN	338, 411	LLE	4 pg	Saisho et al. 1998 [57]
Corticosteroids	Urine	Silica	2-Propanol–hexane	9-AN	370, 470	Enzyme hydrolysis, extraction with 0.5 M NaOH	NR	Neufeld et al. 1998 [50]
F and E	Human plasma	C_18_	ACN: 0.3 mM *ortho*-phosphoric acid (470:530)	9-AN	360, 460	SPE	3.0 ng/mL	Glowka et al. 2009 [45]
Corticosterone	Rat urine	CN	ACN: H_2_O (24.5:75.5)	post-column reaction with sulfuric acid	460, 510	LLE	0.5 pmol	Sudo et al. 1990 [55]
F	Human hair	C_18_	MeOH:H_2_O 60:40	sulfuric acid	360, 480	LLE	1 pg/mg	Gao et al. 2010 [56]
EE2, E2, and BPA	Human urine and aqueous samples	C_18_	ACN:MeOH:H_2_O (30:15:55)	-	280, 310	FPSE	E2: 20 pg/mL EE2: 36 pg/mL BPA: 42 pg/mL	Kumar et al. 2014 [81]
BPA, NP, E2, EE2, and E3	Human urine	C_18_	Gradient elution of ACN and H_2_O	*p*-nitrobenzoyl chloride	E2, E3: 282, 315 BPA, NP, EE2: 228, 316	SPE	BPA and E2: 2.7 μg/L NP: 2.9 μg/L E2 and EE2: 4.6 μg/L E3: 8.3 μg/L	Mao et al. 2004 [42]
E, testosterone, methyltestosterone, bolasterone, testosterone acetate, progesterone	Urine	C_18_	0.01 M Tb(NO_3_)_3_, 0.1 M sodium dodecyl sulfate (SDS), and 20% acetonitrile	-	245, 547	SPE	Down to 100 pg/mL	Amin et al. 1993 [26]
BPA and 8 alkylphenols (4-*sec-*Butylphenol, 2-*tert*-Butylphenol, 3-*tert*-butylphenol, 4-*tert*-butylphenol, 4-*n*-Pentylphenol, 4-*tert*-pentylphenol, 4-*n*-hexylphenol, and 4-*n*-heptylphenol)	Rat plasma and blood	C_18_	MeOH:Water (10:90)	2-(4-carboxyphenyl)-5,6- dimethylbenzimidazole	Derivatized: 336, 440 Native: 275, 315	LLE	BPA: 0.1 pg/mL Alkylphenol: 0.7–10 pg/mL	Katayama et al. 2001 [59]
progesterone and 17-hydroxyprogesterone dehydroepiandrosterone, androstenedione, testosterone and 17-methyltestosterone	Serum from pregnant and non-pregnant women	Wakosil 5C4	Acetonitrile:Water (7:3)	BODIPY FL hydrazide	495, 516	LLE	550–3700 fmol per 10 μL	Katayama et al. 1998 [68]
aldosterone, corticosterone, F, E, dexamethasone, fluocinolone acetonide, triamcinolone and triamcinolone acetonide	Human plasma	C_18_	Water:MeOH (25:75) containing 5 mmol/L tetramethylammonium hydrogen sulphate	CDB	334, 418	LLE	0.06–0.3 pg per 100 μL	Katayama et al. 1992 [58]
BPA	Breast Milk	C_18_	1: ACN:H_2_O:MeOH (72:13:15) 2: ACN:0.1 M acetate buffer (pH 5.5):MeOH (55:12:33)	DIB-Cl	350, 475	SPE then LLE	0.11 ng/mL	Sun et al. 2004 [61]
BPA	Human blood serum and ascitic fluid samples	C_18_	1: ACN: H_2_O:MeOH (72:13:15) 2: ACN:0.1 M Acetate buffer (pH 5.5):MeOH (55:12:33)	DIB-Cl	350, 475	LLE	0.04 ppb	Kuroda et al. 2003 [62]
BPA	Rat brain rat plasma	C_18_	1: ACN: H_2_O:MeOH:Tetrahydrofuran (55:10:35:2.5) 2: ACN:0.1 M Acetate buffer (pH 3.0):MeOH (35:10:55)	DIB-Cl	350, 475	LLE	0.3 ppb in 60 μL rat brain 4.6 ppb in 50 μL rat plasma	Sun et al. 2002 [63]
F, E, PL, PN, 6β-OHF, 6β-OHP and 6β-OHE	Human plasma and urine	Cosmosil 5SL	diethylene dioxide: ethyl acetate:chloroform:*n*-hexane:pyridine (500:100:100:1400:21)	9-AN	360, 460	LLE	F, E, PL and PN: 0.1 ng/mL 6β-OHF and 6β-OHP: 0.5 ng/mL	Shibata et al. 1997 [47]
Corticosterone	Rat serum	C_18_	60% MeOH: 40% 5 mM triethylamine, pH 3.3	-	375, 485	LLE	0.1 ng	Mason et al. 1992 [77]
18-Oxygenated corticosteroids, 18-hydroxycortisol, 18- hydroxycortisone and 18-oxocortisol	Human urine	μBondasphere phenyl	A: 10 mM ammonium acetate: MeOH (50:50) B: ACN	1-AN	370, 470	LLE and SPE	0.1 pmol	Kurosawa et al. 1995 [43]
Cholesterol and sitosterol	Saliva and urine biosamples, Cow milk, and Soybean milk	C8	MeOH:isopropanol:H_2_O (90:5:5)	naproxen acyl chloride	231, 352	LLE	25 nM per 10 μL injected volume	Lin et al. 2007 [79]
Pregnenolone	Rat brain	C_18_	MeOH:H_2_O (9:1)	1-AN	370, 470	SPE	NR	Shimada et al. 1996 [27]
C21 steroids; corticoids	Steroid standards	C_18_	MeOH:H_2_O:cyclodextrin	1-AN	360, 460	NR	NR	Shimada et al. 1991 [44]
Triamcinolone	Human plasma	C_18_	ACN and 0.3 mM ortho-phosphoric acid	9-AN	360, 460	SPE	1 ng/mL	Glowka et al. 2006 [88]
Butane acid-(5-androsten-17-one-3beta-ol)-diester (A1998)	Rat plasma	C_18_	25 mM acetate buffer (pH 3.7):ACN Alfaxalone: (45:55) Pregnanolone: (40:60)	Dansyl hydrazine	332, 516	LLE	10 ng/mL	Visser et al. 2000 [69]
Alfaxalone and pregnanolone	Rat plasma	C_18_	Gradient mixture of ACN and H_2_O	Dansyl Hydrazine	350, 520	-	0.025 μg/mL	Peng et al. 2007 [70]
EED	Oral contraceptive tablets	STAR RP-18e	ACN:H_2_O (47:53)	-	EED: 285, 310	-	EED: 0.0538 μg/ml	Sarafinovska et al. 2006 [12]
EED and drospirenone	Oral contraceptive tablets	STAR RP-18e RP	ACN:H_2_O (47:53)	-	285, 310	-	EED: 0.00065 μg/mL DROSP: 0.0774 μg/mL	Sarafinovska et al. 2009 [13]
EED	Coated tablets	LiChroCART 100RP	ACN:H_2_O (50:50)	-	280, 310	-	EED: 0.02 μg/mL	Silva et al. 2013 [89]
Sodium E1 sulphate, sodium equilin sulphate, E1 and equilin	Raw materials and Pharmaceuticals	5 ODS_2_	TEA phosphate buffer (pH 4.0; 0.05 M):ACN 1— (70:30, *v*:*v*) 2—for unconjugated estrogens: (66:34)	Postcolumn on line photochemical derivatization	280, 410 or 312	-	0.01–1.38 pmol	Gatti et al. 1998 [90]
E1, 17β-Estradiol, E3, BPA, NP, OP	Fish, chicken, aquaculture pond water sample	C_18_	Gradient program of 1: H_2_O: 5% ACN 2: H_2_O: ACN	BCEC-Cl	279, 380	DLLME	0.02–0.07 µg/L	Wu et al. 2015 [29]
E1, E2, and E3	Cow and river Buffalo	C_18_	1: ACN:H_2_O:Formic Acid (40:60:0.4) 2: ACN:H_2_O:Formic Acid (90:10:0.4)	-	280, 310	LLE and SPE	5–10 ng/kg	Shahbazi et al. 2016 [31]
α- and β-Trenbolone	Bovine muscle and liver	C_18_	MeOH:H_2_O (60:40)	-	364, 460	LLE then SPE	bovine muscle: 0.2 ng/g liver: 1.0 ng/g	Yoshioka et al. 2000 [33]
NP, 4-nonylphenol mono-(NP1EO), diethoxylates (NP2EO), BPA, TBP, and OP	Fish and shellfish	Inertsil PH	Gradient program of A: H_2_O B: MeOH	-	275, 300	LLE	NP NP1EO and NP2EO : 2 ng/g BPA, BP and OP: 1 ng/g	Tsuda et al. 2000 [32]
Nonylphenol and its ethoxylates	Fish tissue	Hypersil APS	Hexane:ethanol (98:2)	-	230, 300	Pressurized fluid extraction	4–15 ng/mL	Datta et al. 2002 [28]
E2 and EE2	Poultry litter	C_18_	Gradient program of A: H_2_O B: ACN	-	280, 312	LLE	E2: 4.0 μg/kg EE2: 2.6 μg/kg	Lu et al. 2014 [91]
E2 and EE2	Waste Water	C_18_	Gradient program of A: H_2_O B: ACN	-	282, 306	SPE	2.5 ng/L	Liz et al. 2017 [17]
E3, E2, EE2, HEX, mestranol	Water, sediment	Poroshell 120 EC	H_2_O:ACN 50:50	-	275, 310	SPE	Water: 6–24 ng/L Sediment: 0.1–0.9 ng/g	Perez et al. 2015 [92]
E2, 17α-EE2, and E1	Water	C_18_	Gradient program of A: ACN B: H_2_O acidified at pH 3.6 with glacial acetic acid	-	230, 302	SPE	10 to 1100 ng/L	Patrolecco et al. 2013 [93]
OP, NP, BPA, diethylstilbestrol, E1, EE2, E2, and E3	Wastewater samples	C_18_	Gradient program of A: 5% ACN B: ACN	EASC	262, 430	SPE	0.3–0.7 ng/L	Zhang et al. 2012 [52]
E2 and EE2	Tap, surface and waste water	C_18_	Water:acetonitrile mixture (50:50)	-	280, 310	DLLME	E2: 2.0 ng/L EE2: 6.5 ng/L	Lima et al. 2013 [37]
BPA, 17β-estradiol, and 17α-ethynyl estradiol	Drinking water	LiChro-sorbs RP18	10 mM H_3_PO_4_:55% MeOH (45:55)	-	280, 310	PAC	BPA: 201 ng/L E2: 313 ng/L EE2: 284.5 ng/L	Yoon et al. 2003 [94]
17α- and 17β-Trenbolone	River water	C_18_	Gradient program of MeOH:H_2_O	-	359, 458	SPE	4 ng/L	Durhan et al. 2005 [95]
NP, OP, NP polyethoxylates	municipal wastewater treatment plants	C_18_	Gradient program of H_2_O:ACN	-	229, 310	LLE	OP: 2 ng/L NP: 11 ng/L NPE: 52 ng/L	Snyder et al. 1999 [5]
E2, E3, BPA, and 17β-ethinylestradiol	environmental waters	C_18_	ACN:0.02 mol/L phosphate solution (45:55)	-	227, 315	Synthesized in-tube SPME device	0.006–0.10 ng/mL	Wen et al. 2006 [96]
E2, E3, EE2, 3-methyl ether EE2, NP, OP, POE(1-2) nonyl phenol and BPA	River water	C_18_	Gradient program of Milli-Q H_2_O and ACN	-	230, 290	on-line SPE	20–50 ng/L	Ying et al. 2002 [97]
Endocrine disruptors; BPA and EE2	Environmental water samples	C_18_	MeOH:0.025 mol/L Na_2_HPO_4_ buffer (70:30)	-	220, 315	A poly(acrylamide-vinylpyridine) monolithic capillary column	BPA: 0.064 ng/mL 17α-ethinylestradiol: 0.12 ng/mL	Fan et al. 2005 [98]
Brassinosteroids	Plant: *(Vicia faba* L.)	C_18_	ACN: H_2_O (90:10)	9-Phenanthreneboronic acid	305, 375	LLE	50 pg	Gamoh et al. 1989 [51]
Brassinolide	Arabidopsis thaliana, Daucus carota and Brassica campestris L. leaves’ samples	C_18_	Gradient program of A: H_2_O and ACN B: ACN and 0.1% Formic Acid	-	305, 375	UA-DLLME	8.0 ng/L	Lv et al. 2014 [99]
Brassinolide and castasterone	Pollen of orange (Citrus sinensis Osbeck)	C_18_	ACN:H_2_O (80:20)	Dansylaminophenylboronic acid	345, 515	LLE	NR	Motegi et al. 1994 [72]
Norgestrel, norethindrone, EE2, gestodene, and norethisterone acetate	Meat samples	Hypersil GOLD	Gradient program of (A) H_2_O containing 5% ACN (B)ACN	Fmoc-Cl	250, 395	MSPE	1.4 × 10^3^–8.7 × 10^3^	Qianyu Li et al. 2018 [73]
F and E	Human urine samples	C_18_	ACN:0.3 mM orthophosphoric acid (470: 530)	9-AN	360, 460	LLE	LLOQ: F: 27.6 nmol/L E: 27.7 nmol/L	Kosicka et al. 2018 [49]
FFA	Edible oils and foodstuff	C_8_	Gradient system: A: H_2_O B: ACN/DMF (1:1) C: ACN (100%)	BCETS	279, 380	Supercritical CO_2_ and organic solvent extraction	0.22–1.06 ng/mL	Li et al. 2011 [74]
OP, NP, TBP, BPA, E1, E2, E3	Milk samples	C_18_	Gradient program of A:5% ACN in H_2_O B: ACN	BQEIC	302, 401	LLE	10.5–13.8 ng/L	Liu et al. 2018 [75]
E3, 2-OHE_2_, 17β-E2, 17α-E2, EE_2_, HEX	Dairy products	C_18_	Gradient Flow A: 1 mM formic acid in ACN B: 1 mM formic acid at pH 3.50	-	280, 310/320	HF-LPME	0.23–14.8 μg/kg	Bárbara Socas-Rodríguez et al. 2014 [36]
Zearalenone	Edible oil	C_18_	Gradient program of A: H_2_O, B: ACN		274, 456	SPE	10 μg/kg	Drzymala et al. 2015 [100]
EE2, E1, E2, E3, and progesterone	Drinking water and wastewater samples	C_18_	Gradient program of A: H_2_O/CH_3_CN 90/10 v/v B: CH_3_CN		200, 315	SPE	Drinking water: 1–3.8 ng/L Sewage water: 3.8–7.5 ng/L	Kozłowska-Tylingo et al. 2015 [20]
E3, E2, E1	Human urine	C_18_	Gradient program of A: H_2_O, B: ACN		280, 310	VA-DLLME-FOA	E3: 0.01 ng/mL β-E2: 0.01 ng/mL E1: 0.06 ng/mL	Wang et al. 2015 [82]
17-α-E2, 17-β-E2 benzoate and quinestrol	Environmental water samples	Zorbax Eclipse SB-C_18_	Gradient program of A: ACN, B: H_2_O		265, 311	IF-IHLME	17-α-Estradiol: 0.04 ng/mL E2 and Quinestrol: 0.05 ng/mL	Zhang et al. 2017 [101]
E2 and EE2	Tap water samples	Pursuit 5 C_18_ column	ACN:H_2_O (50:50), with 200 μL of H_3_PO_4_		230, 306	Nanoparticles of graphene oxide/γ-Fe_2_O_3_ as a sorbent for SPE	E2: 2.7 ng/L EE2: 0.8 ng/L	Fernanda Nunes Ferreira et al. 2020 [102]
17-E2 and E3	Water samples	C_18_	H_2_O:MeOH:ACN (50:30:20)		280, 310	ultrasonication assisted DLLME	DLLME-HPLC/FLD: 7.16–69.22 ng/L	Zhang et al. 2020 [14]
E2, 1,3,5(10)-Estratriene-3,17β-diol	Fish and prawn tissue samples	ODS C_18_	H_2_O:MeOH (30:70)		280,310	MISPE	0.023 mg/L	Jiang et al. 2009 [40]
BPA, EE2, 4-t- OP, 4-OP, and 4-NP	River water	C_18_	Gradient program of A: ACN B: H_2_O		277, 307	Disposable pipette extraction (DPX)	BPA, EE2, 4-OP and 4-NP: 0.30 μg/L 4-t-OP: 0.60 μg/L	Gabriela Corazza et al. 2017 [103]
E1 and EE2	Digested sludge	C_18_-PFP	A: H_2_O B: ACN E1: (50:50) EE2 (55:45)		280, 310	ultrasonic liquid extraction	E1: 0.305 μg/g EE2: 0.052 μg/g	Vitória L. Louros et al. 2019 [104]
E2	Milk sample	XDB-C_18_	MeOH:H_2_O (70:30)		280, 310	SPE	0.7 ng/mL	Yanan Yuan et al. 2019 [105]
Nine BPs	milk samples	C_18_	Gradient program of 0.1% formic acid: ACN		230, 305	ultrasonically with acetonitrile and cleaned using the QuEChERS technique.	1.0–3.1 µg/kg	Xiong et al. 2017 [38]
EE	river water samples	5C_18_ MS-II	ACN: 5.0 mM Tris-HNO_3_ buffer, pH 7.4 (60:40)		310, 400	C_18_ SPE disk	7.4 ng/L	Ali et al. 2020 [64]
E3, 17β-estradiol glucuronide, 17β-E2, 17α-E2, 17β-E2-3-methyl ether	wastewater	UPLC C_18_	Gradient program of water with 0.1% of ammonia: ACN		280, 310	Molecularly Imprinted SPE	1.4 to 2.5 ng/mL	Rayco Guedes-Alonso et al. 2015 [41]

Abbreviation: solid phase extraction (SPE), liquid–liquid extraction (LLE), dispersive-liquid–liquid extraction (DLLME), ultrasonic-assisted dispersive liquid–liquid microextraction (UA-DLLME), vortex-assisted dispersive liquid–liquid microextraction method based on floating organic acid droplet (VA-DLLME-FOA), ionic liquid foam floatation coupled with an ionic liquid-based homogeneous liquid–liquid microextraction (IF-IHLME), magnetic solid phase extraction (MSPE), fabric phase sorptive extraction (FPSE), molecularly imprinted solid-phase extraction (MISPE), hollow fiber liquid-phase microextraction (HF-LPME), 4-octylphenol (OP), 4-tert-octylphenol (4-t-OP), 4-nonylphenol (NP), 4-tert-butylphenol (TBP), bisphenol A (BPA), estrone (E1), 17β-estradiol (17β-E2), 17α-estradiol (17α-E2), estriol (E3), free fatty acids (FFA), 17α-ethinylestradiol (EE2), ethinylestradiol (EED), cortisol (F), cortisone (E), 4,40-(1,2-diethylethylene)diphenol (HEX), prednisolone (PL), prednisone (PN), 6β-hydroxycortisol (6β-OHF), 6β-hydroxyprednisolone (6β-OHP) and 6β-hydroxycortisone (6β-OHE), room temperature (RT).
